# Peripheral Antinociception Induced by Carvacrol in the Formalin Test Involves the Opioid Receptor-NO-cGMP-K^+^ Channel Pathway

**DOI:** 10.3390/metabo15050314

**Published:** 2025-05-07

**Authors:** Mario I. Ortiz, Raquel Cariño-Cortés, Eduardo Fernández-Martínez, Victor Manuel Muñoz-Pérez, Gilberto Castañeda-Hernández, Martha Patricia González-García

**Affiliations:** 1 Área Académica de Medicina del Instituto de Ciencias de la Salud, Universidad Autónoma del Estado de Hidalgo, Pachuca 42090, Hidalgo, Mexico; tomedyfm@hotmail.com (E.F.-M.); victor9783@hotmail.com (V.M.M.-P.); 2 Departamento de Farmacología, Centro de Investigación y de Estudios Avanzados del Instituto Politécnico Nacional, Ciudad de México 07360, Mexico; gcastane@cinvestav.mx (G.C.-H.);

**Keywords:** carvacrol, antinociception, nitric oxide, cGMP, K^+^ channels

## Abstract

Background/Objectives: Carvacrol is a naturally occurring phenolic monoterpene that is one of the main constituents of the essential oils of oregano (*Origanum vulgare*) and other herbs. Carvacrol has anti-inflammatory and antinociceptive effects. Carvacrol can activate and inhibit several second messengers and ionic channels at the systemic level. However, there is no evidence of the peripheral antinociception of carvacrol and its mechanism of action. This study was designed to determine whether the opioid receptor-nitric oxide (NO)-cyclic guanosine monophosphate (cGMP)-K^+^ channel pathway is involved in the local antinociception of carvacrol. Methods: Wistar rats were injected with 1% formalin subcutaneously on the dorsal surface of the right hind paw with the vehicle or carvacrol (100–300 µg/paw). To determine whether the opioid receptor-NO-cGMP-K^+^ channel pathway and a biguanide-dependent mechanism are responsible for the local antinociception induced by carvacrol, the effect of the injection (10 min before the 1% formalin injection) with the corresponding vehicles, metformin, naltrexone, NG-L-nitro-arginine methyl ester (L-NAME), 1 H-(1,2,4)-oxadiazolo (4,2-a) quinoxalin-1-one (ODQ), and K^+^ channel blockers on the antinociception induced by local carvacrol (300 µg/paw) was determined. Results: In both phases of the formalin test, carvacrol produced antinociception. Naltrexone, metformin, L-NAME, ODQ, glibenclamide and glipizide (both ATP-sensitive K^+^ channel blockers), tetraethylammonium and 4-aminopyridine (voltage-gated K^+^ channel blockers), and apamin and charybdotoxin (Ca^2+^-activated K^+^ channel blockers) reversed the carvacrol-induced peripheral antinociception. Conclusions: The local peripheral administration of carvacrol produced significant antinociception and activated the opioid receptor-NO-cGMP-K^+^ channel pathway.

## 1. Introduction

Carvacrol, or isothymol (International Union of Pure and Applied Chemistry (IUPAC) name: 2-methyl-5-propan-2-ylphenol), is a naturally occurring phenolic monoterpene derivative of cymene [1]. A colourless to pale-yellow liquid with a characteristic pungent odour, carvacrol is widely used as a flavouring and food additive and is classified as non-toxic for human consumption [1,2]. Carvacrol is one of the major constituents of the essential oils of pepperwort (*Lepidium flavum*), thyme (*Thymus vulgaris*), oregano (*Origanum vulgare*), and other herbs [1,2]. Studies have shown that carvacrol has hepatoprotective, antioxidant, neuroprotective, antidiabetic, anti-inflammatory, and antinociceptive effects [1,2,3,4]. Specific biological effects of carvacrol have been linked to the involvement of nitric oxide (NO), cyclic guanosine monophosphate (cGMP), the ATP-sensitive K^+^ channel, and the voltage-gated K^+^ channel [5,6,7,8]. Other in situ and in vitro studies have demonstrated the ability of carvacrol to activate TRPA1 and TRPV3 channels and to inhibit TRPM7, TRPV1, nicotinic, Ca^2+^, and voltage-gated Na^+^ channels [9,10,11,12,13,14,15,16].

In the antinociceptive activity, carvacrol reduced the nociception produced in several tests [17,18,19,20]. Concerning opioid receptors, there is both positive and negative evidence for their activation by carvacrol in models of nociception [18,20,21]. Carvacrol has also been suggested to activate the opioid receptor-NO-cGMP-K^+^ channel pathway at the systemic level [20]. In this earlier antinociception research, the mechanisms of action of carvacrol were investigated in isolated models and at a systemic level. However, the possible peripheral mechanisms of action of carvacrol, such as the opioid receptor-NO-cGMP-K^+^ channel pathway, have not been investigated [20,22,23,24,25,26,27,28,29,30,31]. The ultimate targets for reducing neuronal or cell excitability are K^+^ and Ca^2+^ channels [32]. The K^+^ channels can be modulated by the NO-cGMP pathway and other second messengers and drugs [32,33]. Therefore, our research was designed to investigate the possible activation of opioid receptors, the NO-cGMP pathway, K^+^ channels, and the effect of metformin on the peripheral antinociceptive effects of carvacrol in the rat formalin test. The discovery of the antinociceptive activity of carvacrol at the local peripheral level allows us to suggest its possible analgesic effect at the clinical level by a topical application, either as a cream or as a carvacrol oil solution. Likewise, the activation of second messengers and ionic channels in the effects of carvacrol will be helpful in examining the probable pharmacological interaction between carvacrol and other drugs involved in activating or blocking the same mechanisms of action, which could result in antagonism or synergism.

## 2. Materials and Methods

### 2.1. Animals

Male Wistar rats aged 7–9 weeks (weight range: 180–220 g) from the breeding facilities of our research centre were used in this study. The rats were housed in a special room with constant temperature (22 + 28 °C) and humidity (50%) levels, 12 h light ± dark cycles (lights on from 7:00 am), and food and water available ad libitum in their cages. Efforts were made to minimise unnecessary handling and suffering and to reduce the number of animals used. Each rat was used in a single experiment and was immediately euthanised in a CO_2_ chamber at the end of the experiment. The Institutional Animal Care and Use Committee (Centro de Investigación y de Estudios Avanzados del Instituto Politécnico Nacional (CINVESTAV.IPN), Ciudad de México, Mexico) approved the study protocol with registration number 0169-15 and approval date 6 April 2016, after which the animals were treated according to the Guiding Principle on Ethical Standards for Investigation in Animals [34].

### 2.2. Drugs

Carvacrol, naltrexone (opioid receptor blocker), metformin (hypoglycaemic biguanide), NG-L-nitro-arginine methyl ester (L-NAME)(an NO synthase inhibitor), 1 H-(1,2,4)-oxadiazolo (4,2-a) quinoxalin-1-one (ODQ)(an NO-sensitive soluble guanylate cyclase (sGC) inhibitor), glibenclamide and glipizide (both ATP-sensitive K^+^ channel blockers; K_ir_6.1-2), tetraethylammonium chloride (TEA) and 4-aminopyridine (4-AP) (both voltage-gated K^+^ channel blockers; K_V_), apamin (a small conductance Ca^2+^-activated K^+^ channel blocker; K_Ca_2.1-3), and charybdotoxin (a big conductance Ca^2+^-activated K^+^ channel blocker; K_Ca_1.1) were obtained from Sigma-Aldrich (Toluca, Mexico). Carvacrol was diluted in a 1% Tween 80 solution. The glipizide, glibenclamide, and ODQ were dissolved in a 20% dimethyl sulfoxide (DMSO) solution. L-NAME, naltrexone, metformin, TEA, 4-AP, apamin, and charybdotoxin were dissolved in a 0.9% saline solution.

### 2.3. Assessment of Nociception in the Formalin Test

The nociceptive and antinociceptive effects were assessed using the rat paw 1% formalin test [26,27,28]. The rats were carefully transported from the vivarium to the laboratory in special polycarbonate rodent cages on the experimental days. Once in the laboratory, the rats were placed in open, transparent Plexiglas observation chambers for 30 min to allow them to adapt to their environment. The rats were then removed for the drug administration. The vehicle or carvacrol (at minute −20), vehicle or blocker (at minute −10), and dilute 1% formalin (at minute zero, in 50 µL each) were injected subcutaneously into the dorsal surface of the right hind paw using a syringe with an 18 mm 30-gauge needle. After the formalin injections, the animals were immediately returned to the observation chambers, a stopwatch was started, and nociceptive behaviour was recorded. Mirrors were placed behind each observation chamber to allow unobstructed examination of the formalin-injected paw. For the present study, nociceptive behaviour was quantified as the number of movements or flinches of the injected paw during 1 min periods, every 5 min, and up to 60 min after the formalin injection. The nociceptive behaviour to the administered formalin was easily detected and characterised as a rapid, brief withdrawal, shake, or flexion of the injected paw. The characteristic formalin-induced paw flinching response in rats is biphasic. An initial acute phase is registered within the first 10 min (phase one), followed by a prolonged tonic response registered from 15 to 60 min (phase two). The area under the curve was estimated for both test phases, and a significant reduction in area was interpreted as an antinociceptive effect.

### 2.4. Study Design

To determine the local peripheral antinociceptive effect of carvacrol, rats in several independent groups were administered either the vehicle (1% Tween solution) or increased doses of carvacrol (30–300 µg/paw). These injections were made subcutaneously in the dorsal region of the right hind paw of the rat 20 min before the administration of 50 µL of 1% formalin to the same site on the paw (Table 1). To determine that the effect of carvacrol was localised to the injection site (excluding systemic effects), a dose of 300 µg/paw of carvacrol was administered to the contralateral (CL, left) paw 20 min before 1% formalin was administered to the ipsilateral (IL, right) paw, and the response was assessed (Table 1).

To investigate the possible involvement of opioid receptors, a metformin-dependent mechanism, and NO and cGMP production in the peripheral antinociceptive effects of carvacrol, independent groups of rats were first treated with 300 µg/paw of carvacrol or its vehicle (1% Tween solution) in the dorsal region of the right hind paw. Ten minutes later, the corresponding vehicles (saline or 20% DMSO solution) or naltrexone (50 µg/paw), metformin (400 µg/paw), L-NAME (100 µg/paw), and ODQ (100 µg/paw) were administered to the same site on the paw of the rats (Table 1). Ten minutes after the administration of the blockers or their vehicles, the 1% formalin solution was administered, and the twitches were scored as described above (Table 1).

To determine whether carvacrol-induced antinociception is mediated by the activation of K^+^ channels, independent groups of rats were first treated with carvacrol (300 µg/paw) or its vehicle (1% Tween solution) in the dorsal region of the right hind paw. Ten minutes after the carvacrol treatment, the animals were treated in the same paw with the appropriate vehicle (saline or 20% DMSO solution) or glibenclamide (100 µg/paw), glipizide (100 µg/paw), apamin (2 µg/paw), charybdotoxin (2 µg/paw), 4-AP (100 µg/paw), or TEA (100 µg/paw). Ten minutes after the vehicle or blocker administration, the formalin was administered to the same paw, and flinching responses were recorded (Table 1). Pilot studies conducted in our laboratory and previous reports [20,22,23,26,27,28,29,30,31] were used to determine the peripheral administration dosages and drug release schedules. Each dose of carvacrol and each dose of blockers were injected in a 50 µL vehicle volume. The potential adverse effects were assessed in rats from each experimental group.

### 2.5. Statistics and Data Analysis

Every experimental outcome is shown as the mean ± standard error of the mean (SEM) for each group of five animals. Curves were constructed by plotting the number of flinches versus time. The area under the curve (AUC) of the number of flinches versus time was calculated using the trapezoidal rule. Both phases of the formalin test are reported. The results were analysed by a one-way analysis of variance (ANOVA) using Statistical Package for the Social Sciences (SPSS) version 20 for Windows. Dunnett’s test was then used to compare the differences among the different groups. The differences were considered to be statistically significant if *p* < 0.05.

## 3. Results

### 3.1. The Antinociceptive Properties of Carvacrol

**Figure 1:**
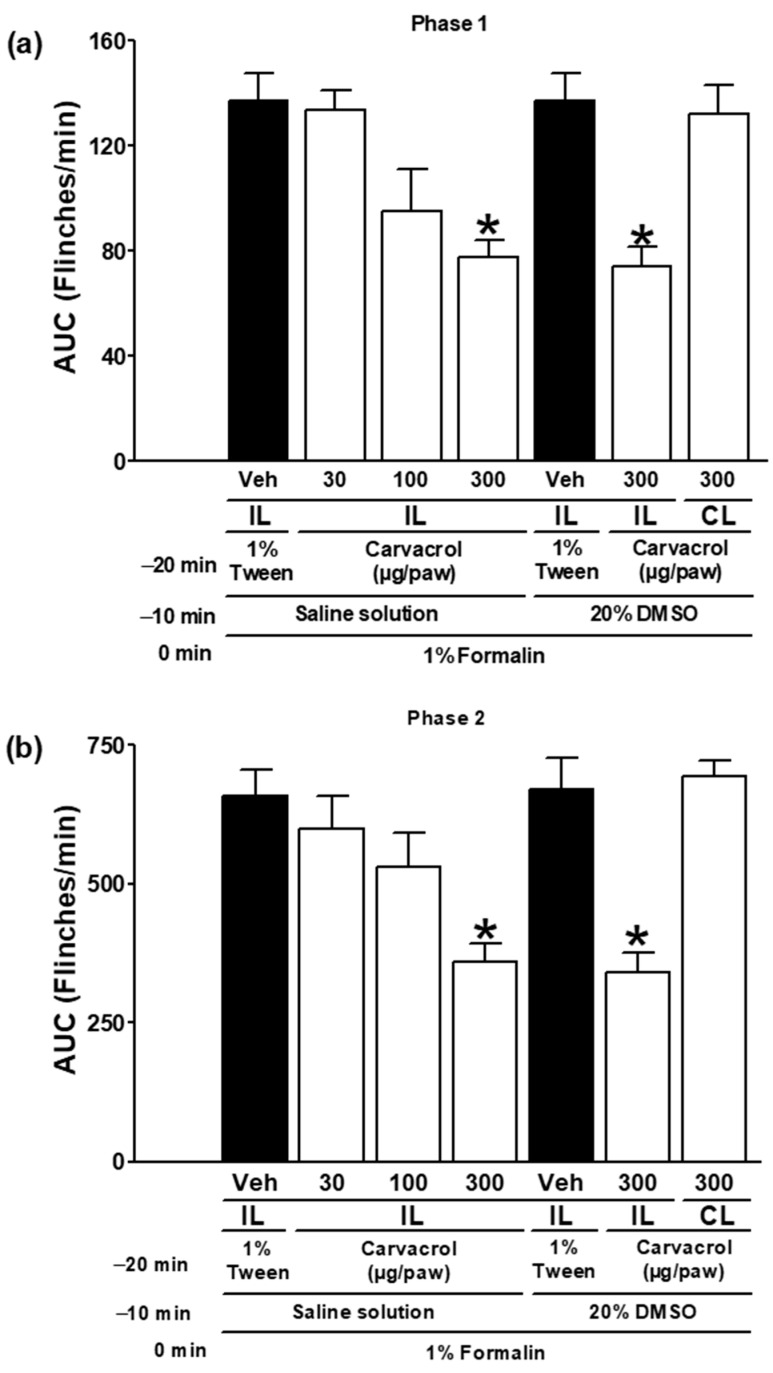
The antinociceptive effect of carvacrol in (**a**) phase one and (**b**) phase two of the 1% formalin test. At minute −20, four groups of rats were injected subcutaneously (sc) in the dorsal region of the right paw (ipsilateral, IL) with carvacrol (30, 100, and 300 µg/paw) or its vehicle (1% Tween solution). At minute −10, the same rats received saline (antagonist vehicle) sc in the same area of the right paw (IL). At minute zero, they received 1% formalin sc in the same area of the right paw (IL), and their nociceptive behaviour was observed for 60 min. Two other groups of independent rats were treated at minute −20 with carvacrol (300 µg/paw) or its vehicle (1% Tween solution) sc in the dorsal region of the right paw (IL). The same rats were treated at minute −10 with a 20% DMSO solution (IL, antagonist vehicle) and at minute zero with 1% formalin (IL), and nociception was quantified for 60 min. Finally, in an independent group of rats, carvacrol (300 µg/paw) was administered sc to the dorsal region of the left paw (contralateral, CL) at minute −20. At minute −10, rats received 20% DMSO solution sc in the right paw (IL), and at minute zero, 1% formalin in the right paw (IL), and nociception was quantified. The data are presented as the AUC of the number of flinches versus time for both phases. Each point represents the mean ± SEM of five rats. * Significantly different from the vehicle group (*p* < 0.05) by a one-way ANOVA followed by Dunnett’s test.

The local peripheral injection of 1% formalin induced a paw-flinching behaviour, which indicates nociception. The local peripheral administration of carvacrol to the right hind paw significantly reduced the number of formalin-induced flinches, demonstrating a significant antinociceptive effect (*p* < 0.05; Figure 1a,b). The antinociceptive effect of carvacrol was dose-dependent. The nociception produced by the formalin injection into the right paw was not significantly altered by the carvacrol administration to the left (contralateral) paw (*p* > 0.05; Figure 1a,b). The carvacrol-induced antinociception was statistically significant in both phases of the 1% rat formalin test (*p* < 0.05; Figure 1a,b).

### 3.2. Effect of Naltrexone, Metformin, and NO-cGMP Pathway Inhibitors on Carvacrol Antinociception

**Figure 2:**
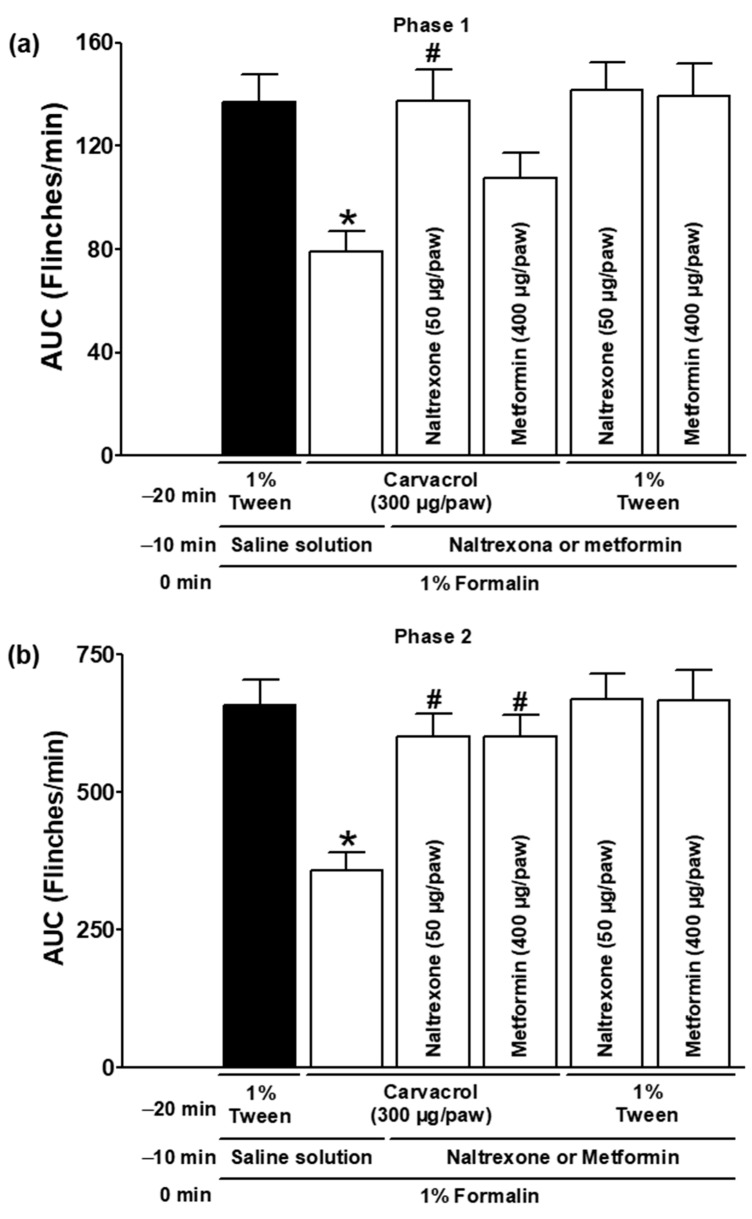
The effect of naltrexone and metformin on the local peripheral antinociceptive activity of carvacrol during phases one (**a**) and two (**b**) of the formalin assays. At minute −20, three groups of rats were injected sc with carvacrol (300 µg/paw) in the dorsal region of the right paw. At minute −10, one group of the same rats received a subcutaneous injection of naltrexone (50 µg/paw), another group received metformin (400 µg/paw), and the last group received an antagonist vehicle (saline) in the same region of the right paw. At minute zero, 1% formalin was applied to the same site on the right paw of all rats, and nociception was quantified. In two other independent groups of rats, a 1% Tween solution was applied to the dorsal region of the right paw at minute −20. Naltrexone (50 µg/paw) was administered to one group of rats and metformin (400 µg/paw) to the other group at the same site at minute −10, and 1% formalin was administered to all rats at minute zero; nociception was then quantified. The data are expressed as the AUC of the number of flinches versus time in both phases. Each point represents the mean ± SEM of five animals. * Significantly different from the vehicle group (*p* < 0.05; this group, represented by the black bar, received the 1% Tween solution at −20 min, saline at −10 min, and formalin at zero min); # significantly different from the carvacrol group (*p* < 0.05) as determined by the one-way ANOVA followed by Dunnett’s test.

Naltrexone modified the carvacrol-induced antinociception in both phases of the formalin test, demonstrating the involvement of opioid receptors (*p <* 0.05; Figure 2a,b). The local administration of metformin significantly reduced the carvacrol-induced antinociception in phase two (*p <* 0.05; Figure 2b) but not in phase one (*p* > 0.05; Figure 2a) of the formalin test. The formalin-induced nociceptive responses were not significantly altered by naltrexone or metformin when administered with the carvacrol vehicle (*p* > 0.05; Figure 2a,b).

**Figure 3:**
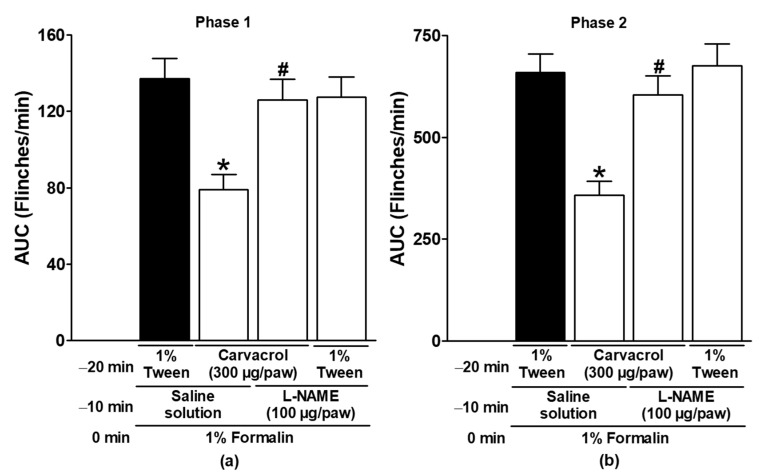
The effect of L-NAME on the local peripheral antinociceptive activity of carvacrol during phases one (**a**) and two (**b**) of the formalin assays. At minute −20, two groups of rats were injected sc with carvacrol (300 µg/paw) in the dorsal region of the right paw. At minute −10, one group of the same rats received subcutaneous injections of L-NAME (100 µg/paw), and the other group received the vehicle (saline) in the same region of the right paw. At minute zero, 1% formalin was applied to the same site on the right paw of all of the rats, and nociception was quantified. In another independent group of rats, a 1% Tween solution was applied sc to the dorsal region of the right paw at minute −20. L-NAME (100 µg/paw) was administered to the same site at minute −10 and 1% formalin at minute zero, and nociception was then quantified. The data are expressed as the AUC of the number of flinches versus time in both phases. Each point represents the mean ± SEM of five animals. * Significantly different from the vehicle group (*p* < 0.05; this group, represented by the black bar, received the 1% Tween solution at −20 min, saline at −10 min, and formalin at zero min); # significantly different from the carvacrol group (*p* < 0.05) as determined by a one-way ANOVA followed by Dunnett’s test.

**Figure 4:**
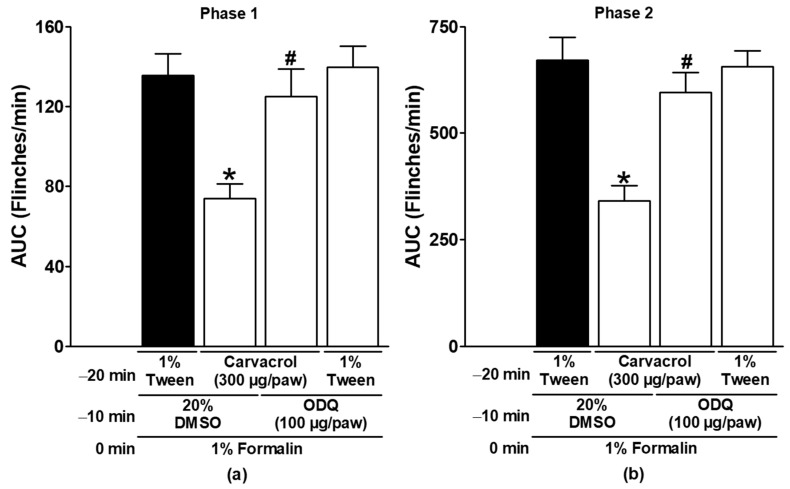
The effect of ODQ on the local peripheral antinociception of carvacrol during phases one (**a**) and two (**b**) of the formalin assays. At minute −20, two groups of rats were injected sc with carvacrol (300 µg/paw) in the dorsal region of the right paw. At minute −10, one group of the same rats received subcutaneous injections of ODQ (100 µg/paw), and the other group received an antagonist vehicle (20% DMSO) in the same region of the right paw. At minute zero, 1% formalin was applied to the same site on the right paw of all of the rats, and nociception was quantified. In another independent group of rats, the 1% Tween solution was applied sc to the dorsal region of the right paw at minute −20. ODQ (100 µg/paw) was applied to the same site at minute −10 and 1% formalin at minute zero, and nociception was then quantified. The data are expressed as the AUC of the number of flinches versus time in both phases. Each point represents the mean ± SEM of five animals. * Significantly different from vehicle group (*p* < 0.05; this group, represented by the black bar, received the 1% Tween solution at −20 min, 20% DMSO at −10 min, and formalin at zero min); # significantly different from the carvacrol group (*p* < 0.05) as determined by the one-way ANOVA followed by Dunnett’s test.

In both formalin test phases, the antinociceptive effects of carvacrol were significantly reduced by the local peripheral injection of L-NAME and ODQ (*p* < 0.05; Figure 3 and Figure 4). These results demonstrate the peripheral generation of NO and cGMP in the antinociception induced by carvacrol. Neither L-NAME nor ODQ significantly altered the formalin-induced nociceptive responses when administered with the carvacrol vehicle (*p* > 0.05; Figure 3 and Figure 4).

### 3.3. Effect of K^+^ Channel Blockers on Carvacrol Antinociception

**Figure 5:**
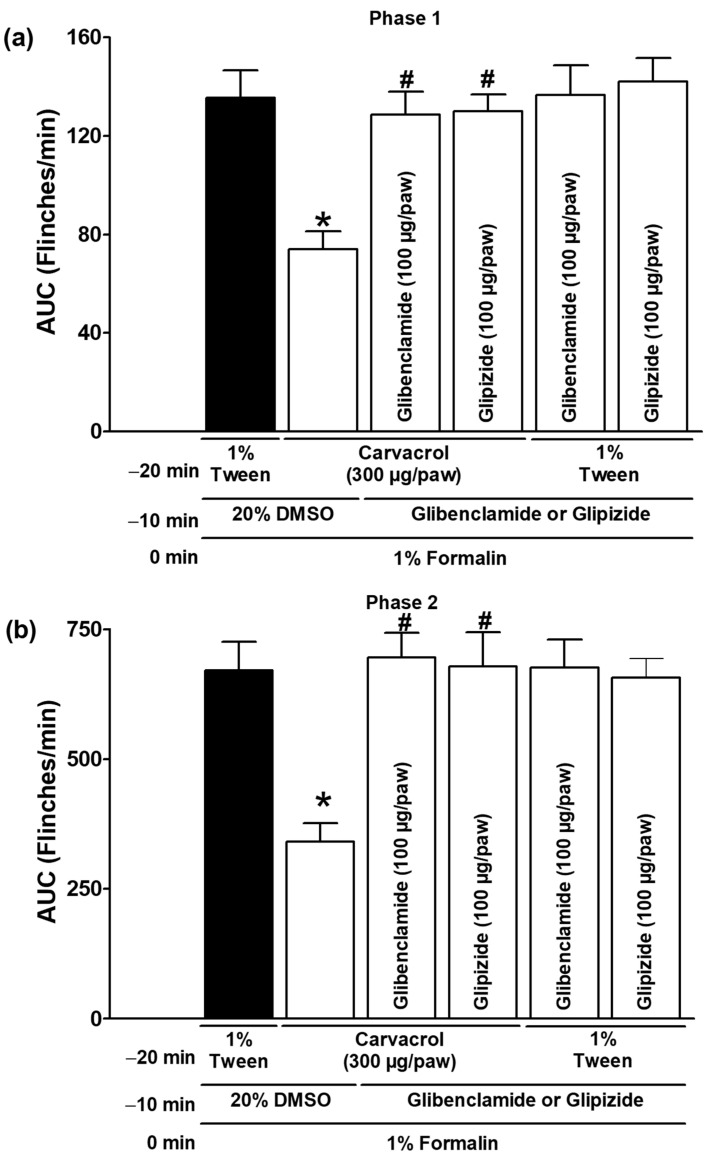
The effect of glibenclamide and glipizide on the local peripheral antinociceptive activity of carvacrol during phases one (**a**) and two (**b**) of the formalin assays. At minute −20, three groups of rats were injected sc with carvacrol (300 µg/paw) in the dorsal region of the right paw. At minute −10, one group of the same rats received a subcutaneous injection of glibenclamide (100 µg/paw), another group received glipizide (100 µg/paw), and the last group received an antagonist vehicle (20% DMSO) at the same site on the right paw. At minute zero, 1% formalin was applied to the same site on the right paw of all of the rats, and nociception was quantified. In two independent groups of rats, the 1% Tween solution was applied sc to the dorsal region of the right paw at minute −20. Glibenclamide (100 µg/paw) was administered to one group of rats and glipizide (100 µg/paw) to the other group at the same site at minute −10, and 1% formalin was administered to all rats at minute zero; nociception was then quantified. The data are expressed as the AUC of the number of flinches versus time in both phases. Each point represents the mean ± SEM of five animals. * Significantly different from vehicle group (*p* < 0.05; this group, represented by the black bar, received the 1% Tween solution at −20 min, 20% DMSO at −10 min, and formalin at zero min); # significantly different from the carvacrol group (*p* < 0.05) as determined by the one-way ANOVA followed by Dunnett’s test.

**Figure 6:**
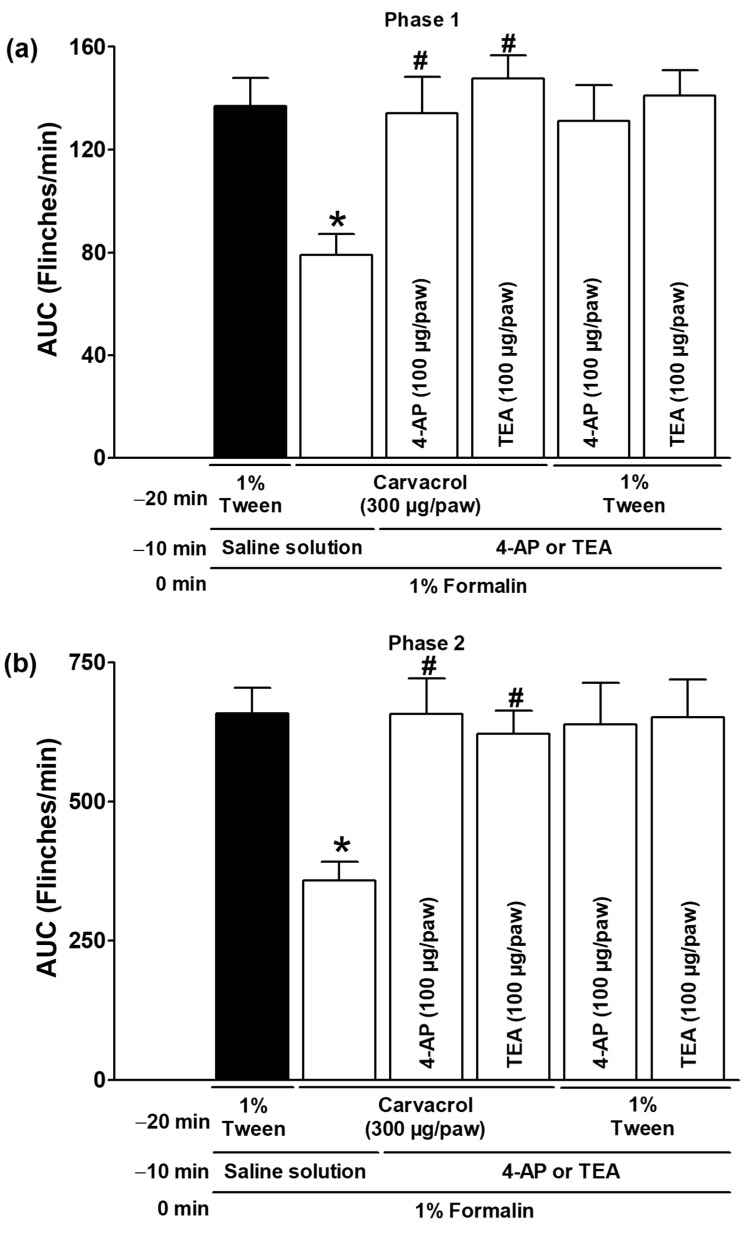
The effect of 4-AP and TEA on the local peripheral antinociceptive activity of carvacrol during phases one (**a**) and two (**b**) of the formalin assays. At minute −20, three groups of rats were injected sc with carvacrol (300 µg/paw) in the dorsal region of the right paw. At minute −10, one group of the same rats received subcutaneous injections of 4-AP (100 µg/paw), another group received TEA (100 µg/paw), and the last group received an antagonist vehicle (saline) in the same region of the right paw. At minute zero, 1% formalin was injected at the same site on the right paw, and nociception was assessed. In two independent groups of rats, the 1% Tween solution was injected into the dorsal region of the right paw at minute −20. 4-AP (100 µg/paw) was administered to one group of rats and TEA (100 µg/paw) to the other group at the same site at minute −10, and 1% formalin was administered to all rats at minute zero; nociception was then quantified. The data are expressed as the AUC of the number of flinches versus time in both phases. Each point represents the mean ± SEM of five animals. * Significantly different from the vehicle group (*p* < 0.05; this group, represented by the black bar, received the 1% Tween solution at −20 min, saline at −10 min, and formalin at zero min); # significantly different from the carvacrol group (*p* < 0.05) as determined by the one-way analysis of variance (ANOVA) followed by Dunnett’s test.

**Figure 7:**
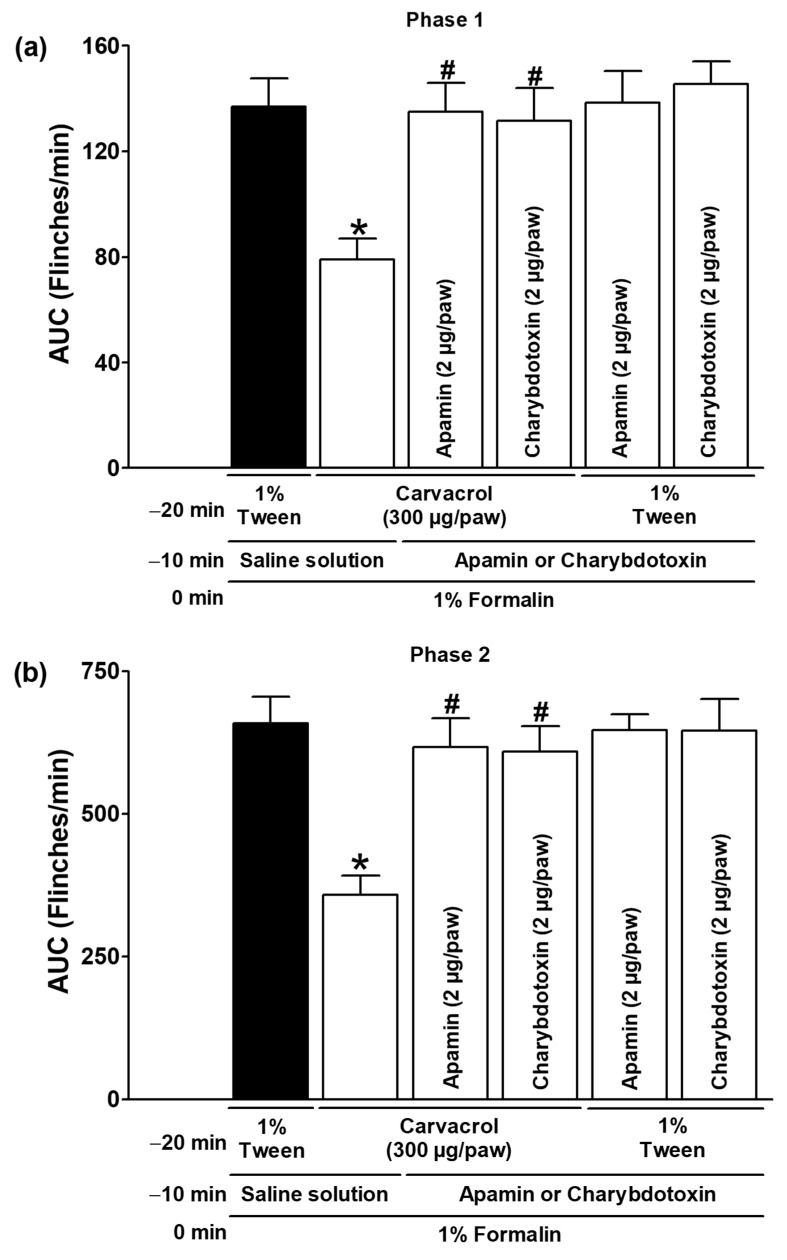
The responses of apamin and charybdotoxin to the local peripheral antinociceptive effect of carvacrol during phases one (**a**) and two (**b**) of the formalin assays. At minute −20, three groups of rats were injected sc with carvacrol (300 µg/paw) in the dorsal region of the right paw. At minute −10, one group of the same rats received subcutaneous injections of apamin (2 µg/paw), another group received charybdotoxin (2 µg/paw), and the last group received an antagonist vehicle (saline) in the same region of the right paw. At minute zero, 1% formalin was injected into the same site on the right paw of all of the rats, and nociception was assessed. In two independent groups of rats, the 1% Tween solution was injected into the dorsal region of the right paw at minute −20. Apamin (2 µg/paw) was administered to one group of rats and charybdotoxin (2 µg/paw) to the other group at the same site at minute −10, and 1% formalin was administered to all rats at minute zero; nociception was then quantified. The data are expressed as the AUC of the number of flinches versus time in both phases. Each point represents the mean ± SEM of five animals. * Significantly different from the vehicle group (*p* < 0.05; this group, represented by the black bar, received the 1% Tween solution at −20 min, saline at −10 min, and formalin at zero min); # significantly different from the carvacrol group (*p* < 0.05) as determined by the one-way ANOVA followed by Dunnett’s test.

Glipizide and glibenclamide, administered subcutaneously, significantly reduced the antinociceptive effects of carvacrol in both test phases, suggesting that carvacrol activates ATP-sensitive K^+^ channels as part of its mechanism of action (*p* < 0.05; Figure 5a,b). The local peripheral injection of 4-AP and TEA significantly reduced the carvacrol-induced antinociceptive effects during both phases of the formalin test, indicating the activation of voltage-gated K^+^ channels by carvacrol (*p* < 0.05; Figure 6a,b). In both test phases, the antinociceptive effects of carvacrol were significantly reduced by the subcutaneous peripheral injection of apamin and charybdotoxin, indicating that carvacrol stimulates the Ca^2+^-activated K^+^ channel (*p* < 0.05; Figure 7a,b). K^+^ channel blockers did not significantly alter the formalin-induced nociceptive behaviour when administered with the carvacrol vehicle (1% Tween solution) (*p* > 0.05; Figure 5, Figure 6 and Figure 7).

## 4. Discussion

There are currently a variety of herbal preparations used as complementary or alternative medicines. These herbal preparations contain various chemical substances that produce a pharmacological effect (either therapeutic or undesirable). Carvacrol is a phenol with multiple biological activities, including antinociceptive and anti-inflammatory effects [4,17,18,19,20]. Both phase 1 (non-inflammatory phase) and phase 2 (inflammatory phase) of the formalin test were significantly reduced by the local peripheral administration of carvacrol in the current study (Figure 1). The carvacrol treatment was unsuccessful in the contralateral paw, so these effects were confined to the injection site (Figure 1). During phase 1 of the formalin test, there is evidence of TRPA1 activation in primary afferent neurons. Peripheral depolarisation and inflammatory sensitisation in spinal cord dorsal horn tissue are caused by the activation of TRPA1 channels and the synthesis of prostaglandins and interleukins during phase 2 of the formalin test [35,36]. Carvacrol is a blocker of cation channels and an activator of TRPV3 channels [9,10,11,12,13,14,15,16]. Carvacrol activated and subsequently desensitised TRPA1 channels in HEK293 cells [9]. In this sense, the probable inactivation of TRPA1 and voltage-gated cation channels produced by carvacrol in its antinociceptive effects determined in the present study cannot be excluded [10,14,16]. As noted above, carvacrol inhibits inflammatory pain in several experimental models, including the second phase of the formalin test. However, a previous study found that carvacrol was unable to block the arachidonic inflammatory cascade (involving the production of prostaglandins, leukotrienes, or epoxyeicosatrienoic acid derivatives) in isolated rat aortic rings [7]. Therefore, further studies in other experimental models are needed to determine the ability of carvacrol to inhibit pro-inflammatory mediators such as prostaglandins, cytokines, chemokines, proteases, neuropeptides, and growth factors.

Opioid drugs and some phytoconstituents can reduce both phases of the formalin test [29,30,36]. On the other hand, nonsteroidal anti-inflammatory drugs (NSAIDs) suppress only the second phase [26,31,36,37]. In the present study, the local peripheral naltrexone injection significantly reversed carvacrol-induced antinociception (Figure 2). This result is consistent with the ability of naloxone to block the systemic antinociception induced by the essential oils of *Ziziphora clinopodioides* (oil containing 65.2% carvacrol and 34.8% other phytochemicals) and *Thymus persicus* (oil containing 32.2% carvacrol and 67.8% other phytochemicals) in the formalin test [20,21]. In contrast, opioid receptors are not involved in the systemic antinociceptive effect of carvacrol on both phases of the formalin test in mice [18]. These discrepancies are likely due to the different species used and the different routes and times of the administration of the drugs.

NO is a gas synthesised in tissues by the enzyme NO synthase (NOS), which uses the semi-essential amino acid L-arginine to form citrulline and NO in a 1:1 ratio. The released NO travels to target cells and activates the enzyme sGC to synthesize cGMP from guanosine triphosphate (GTP). cGMP acts as a second messenger, directly or through protein kinase G (PKG) activation, to open potassium channels and modulate Ca^2+^ channels. The action of cGMP is terminated by its inactivation employing a phosphodiesterase enzyme [38,39,40]. The production of NO and cGMP is involved in immunity, inflammation, platelet aggregation, nociception, smooth muscle relaxation, and other pathological and physiological processes [38,39,40]. In some models of nociception, the production of NO and cGMP is involved in the antinociceptive effects of several drugs, including NSAIDs, phytoconstituents, opioids, and neuromodulators [20,22,23,24,25,26,27,28,29]. The local peripheral administration of L-NAME, an NO synthesis inhibitor, and ODQ, an NO-sensitive sGC inhibitor [38,39,40], significantly reversed the antinociceptive effect of carvacrol in the present study (Figure 3 and Figure 4). These results are consistent with the production of NO and cGMP in the gastroprotection produced by carvacrol in rats and the antinociception produced by the essential oils of *Lippia origanoides* (oil containing 53.9% carvacrol and 46.1% other phytochemicals) and *T. persicus* aerial parts [5,8,20].

In contrast, neither L-NAME nor ODQ blocked the endothelium-dependent relaxation of carvacrol in rat aorta [7]. In the latter case, the discrepancy is mainly due to the difference in the experimental model. To our knowledge, the ability of locally administered carvacrol to trigger the NO-cGMP pathway and produce peripheral antinociception has never been documented.

Potassium channels are essential membrane proteins that control the transport of K^+^ ions across the membrane. In excitable cells, such as neurons, the K^+^ channel activity always reduces excitability by moving the membrane potential away from the firing threshold (hyperpolarization) [32]. According to their structure and functional properties, there are four different types of K^+^ channels [32]. Many drugs, intracellular calcium, membrane depolarisation, and second messengers, such as NO and cGMP, can modulate K^+^ channels [32,33]. Abundant evidence has demonstrated the involvement of some K^+^ channels in systemic or local antinociception induced by various types of analgesic and non-analgesic drugs [22,24,25,26,27,28,29,30,31,41]. There is no hierarchy of potassium channel involvement in the antinociception and antihyperalgesia of different drugs. Although ATP-sensitive K^+^ channels have been identified as the primary participants in the peripheral and central antinociception of analgesics and neuromodulators, there is also strong evidence for the important role of voltage-gated and Ca^2+^-activated K^+^ channels [22,24,25,26,27,28,29,30,31,41,42,43]. The peripherally administered glibenclamide and glipizide were able to counteract the effects of carvacrol in the current study (Figure 5), suggesting that carvacrol can activate ATP-sensitive K^+^ channels in addition to the NO-cGMP pathway to produce its peripheral antinociceptive effect. These results with sulfonylureas are consistent with the involvement of the ATP-sensitive K^+^ channel in the gastroprotection produced by carvacrol, the prevention of apoptosis on rat testis by carvacrol, and the antinociception of the essential oil of *T. persicus* aerial parts in the formalin test [5,6,20]. The voltage-gated K^+^ channel inhibitors 4-AP and TEA, as well as the Ca^2+^-activated K^+^ channel blockers apamin and charybdotoxin, prevented the effects of carvacrol in other experimental groups in the current study (Figure 6 and Figure 7), indicating that these channels are activated in its peripheral antinociception. These results are consistent with the carvacrol-induced relaxation of isolated rat aortic rings by the activation of voltage-gated K^+^ channels and the relaxant effects of *L. origanoides* essential oil in tracheal tissue by the activation of voltage-gated and Ca^2+^-activated K^+^ channels [7,8].

Liver tissue is the primary site of action for the hypoglycaemic drug metformin. Metformin is a first-line treatment for type 2 diabetes. There is evidence that metformin induces hypoglycaemic and metabolic effects by activating AMP-activated protein kinase (AMPK) [44,45]. In addition, AMPK activation inhibits the phosphorylation and activation of other signalling mediators in the nociceptive pathway, leading to antinociceptive effects in some animal models [44,45]. Metformin also inhibits microglial activation and attenuates microglial-mediated neuroinflammation through AMPK activation. The latter effect has been linked to the suppression or reduction of morphine tolerance [44]. However, some studies have linked metformin to the reversal of the antinociceptive effects of some drugs. In the present study, metformin could reverse the antinociceptive effect of carvacrol during phase 2 (Figure 2). This latter result is consistent with the ability of metformin to block the local peripheral antinociceptive effects produced by citral, pamabrom, diclofenac, indomethacin, and metamizole [30,31,37,46]. The exact mechanism by which metformin blocks the antinociceptive effects of carvacrol is currently unknown, as most of the molecular mechanisms demonstrated for metformin appear to be related to antinociceptive and anti-inflammatory effects [44,45]. It is important to note that most of the observed antinociceptive and analgesic effects of metformin are due to its systemic administration [44,45]. Therefore, preclinical and clinical studies are recommended to assess the potential for peripheral local beneficial or harmful metformin interactions with terpenoids or NSAIDs.

Pain management for skin lesions or joint and muscle disorders is achieved by the systemic administration of anti-inflammatory analgesics or neuromodulators [47]. However, topical or dermal medications in creams, gels, patches, or solutions are also good therapeutic options for pain relief in sprains or osteoarthritis of the hand, wrist, foot, ankle, and other sites [48]. In this regard, the present study has shown that carvacrol has local peripheral antinociception. This result suggests its potential clinical analgesic effect by topical application, either as a cream or as a carvacrol oil solution. On the other hand, the activation of the opioid receptor-NO-cGMP-K^+^ channel pathway in the effects of carvacrol will be useful in considering the probable pharmacological interaction between carvacrol and drugs that activate or block the same pathway, which could result in antagonism or potentiation. In this sense, further preclinical and clinical studies are required to evaluate the efficacy and safety of carvacrol in antinociception and pain relief.

## 5. Conclusions

The local peripheral administration of carvacrol produced significant antinociception in both phases of the 1% rat formalin test. The mechanism of action of the peripheral antinociceptive activity of carvacrol involves the activation of the opioid receptor-NO-cGMP pathway and K^+^ channels. Metformin was also able to interfere with carvacrol-induced antinociception at the peripheral level. The antinociceptive properties of carvacrol make it a possible clinical option for topical administration to relieve local, muscular, or joint pain. Given the mechanism of action of carvacrol, it can be administered with other drugs with different mechanisms of action to potentiate its antinociceptive or analgesic effects.

## Figures and Tables

**Table 1:** Timeline of drug and formalin administration schedules.

−20 min Administrations in: IL = Right Paw CL = Left Paw	−10 min Administrations in the Right Paw	Zero Minute Injection of 1% Formalin in the Right Paw	Figures
1% Tween solution (IL)	Saline solution	✓	Figure 1
1% Tween solution (IL)	20% DMSO solution	✓	
Carvacrol (30–300 µg/paw, IL)	Saline solution	✓	
Carvacrol (300 µg/paw, IL)	20% DMSO solution	✓	
Carvacrol (300 µg/paw, CL)	20% DMSO solution	✓	
Carvacrol (300 µg/paw, IL)	Naltrexone (400 µg/paw)	✓	Figure 2
Carvacrol (300 µg/paw, IL)	Metformin (400 µg/paw)	✓	
1% Tween solution (IL)	Naltrexone (400 µg/paw)	✓	
1% Tween solution (IL)	Metformin (400 µg/paw)	✓	
Carvacrol (300 µg/paw, IL)	L-NAME (100 µg/paw)	✓	Figure 3
1% Tween solution (IL)	L-NAME (100 µg/paw)	✓	
Carvacrol (300 µg/paw, IL)	ODQ (100 µg/paw)	✓	Figure 4
1% Tween solution (IL)	ODQ (100 µg/paw)	✓	
Carvacrol (300 µg/paw, IL)	Glibenclamide (100 µg/paw)	✓	Figure 5
Carvacrol (300 µg/paw, IL)	Glipizide (100 µg/paw)	✓	
1% Tween solution (IL)	Glibenclamide (100 µg/paw)	✓	
1% Tween solution (IL)	Glipizide (100 µg/paw)	✓	
Carvacrol (300 µg/paw, IL)	4-AP (100 µg/paw)	✓	Figure 6
Carvacrol (300 µg/paw, IL)	TEA (100 µg/paw)	✓	
1% Tween solution (IL)	4-AP (100 µg/paw)	✓	
1% Tween solution (IL)	TEA (100 µg/paw)	✓	
Carvacrol (300 µg/paw, IL)	Apamin (2 µg/paw)	✓	Figure 7
Carvacrol (300 µg/paw, IL)	Charybdotoxin (2 µg/paw)	✓	
1% Tween solution (IL)	Apamin (2 µg/paw)	✓	
1% Tween solution (IL)	Charybdotoxin (2 µg/paw)	✓	

## Data Availability

The raw data supporting the conclusions of this article will be made available by the author, without undue reservation.

## Abbreviations

The following abbreviations are used in this manuscript:

AMPK	AMP-activated protein kinase
ANOVA	Analysis of variance
ATP	Adenosine triphosphate
AUC	Area under the curve
cGMP	Cyclic Guanosine monophosphate
CINVESTAV.IPN	Centro de Investigación y de Estudios Avanzados del Instituto Politécnico Nacional
CL	Contralateral
DMSO	Dimethyl sulfoxide
GTP	Guanosine triphosphate
IL	Ipsilateral
IPN	Instituto Politécnico Nacional
IUPAC	International Union of Pure and Applied Chemistry
L-NAME	NG-L-nitro-arginine methyl ester
MDPI	Multidisciplinary Digital Publishing Institute
NO	Nitric Oxide
NOS	Nitric Oxide synthase
NSAIDs	Nonsteroidal anti-inflammatory drugs
ODQ	1 H-(1,2,4)-oxadiazolo (4,2-a) quinoxalin-1-one
PKG	Protein kinase G
sc	Subcutaneously
SEM	Standard error of the mean
SPSS	Statistical Package for the Social Sciences
sGC	Soluble guanylate cyclase
TEA	Tetraethylammonium chloride
VEH	Vehicle
IUPAC	The International Union of Pure and Applied Chemistry
4-AP	4-aminopyridine
